# Central Tendon-Splitting Approach and Double Row Suturing for the Treatment of Insertional Achilles Tendinopathy

**DOI:** 10.1155/2019/4920647

**Published:** 2019-08-21

**Authors:** Ze Zhuang, Yang Yang, Kishor Chhantyal, Jianning Chen, Guohui Yuan, Yirong Ni, Dezhao Liu, Dehai Shi

**Affiliations:** ^1^Departments of Joint Surgery and Orthopedic Trauma, The Third Affiliated Hospital of Sun Yat-sen University, Guangzhou 510630, Guangdong, China; ^2^Departments of Spine Surgery, The Third Affiliated Hospital of Sun Yat-sen University, Guangzhou 510630, Guangdong, China; ^3^Departments of Pathology, The Third Affiliated Hospital of Sun Yat-sen University, Guangzhou 510630, Guangdong, China; ^4^MOE Key Laboratory of Laser Life Science & SATCM Third Grade Laboratory of Chinese Medicine and Photonics Technology, College of Biophotonics, South China Normal University, Guangzhou 510631, China; ^5^Departments of Anesthesiology, The Third Affiliated Hospital of Sun Yat-sen University, Guangzhou 510630, Guangdong, China

## Abstract

**Background:**

To assess the clinical outcomes of central tendon-splitting approach and double row anchor suturing for the treatment of insertional Achilles tendinopathy.

**Methods:**

28 patients (28 feet) diagnosed with insertional Achilles tendinopathy were included in this study. The inclusions were symptom of hindfoot pain around the insertion of the Achilles tendon, radiographic demonstration of calcification, or degeneration of the Achilles tendon, showing no symptom improvement even after standard nonsurgical treatment for more than six months. The X-ray revealed that patients had obvious posterior superior calcaneal exostosis with the possibility of friction with the Achilles tendon or intratendinous calcification. Surgical correction by the central tendon-splitting approach and double row Achilles tendon suturing was performed. The ankles were immobilized with plaster for four weeks postoperatively. The American Orthopaedic Foot and Ankle Society (AOFAS) score and visual analogue score (VAS) were assessed preoperatively and at 2 years postoperatively. At final follow-up, the Manchester-Oxford Foot Questionnaire (MOXFQ) as patient-reported outcome measures (PROMs) was also evaluated.

**Results:**

No complication, including postoperative wound infection and tendon rupture, was not found. All the patients resumed their daily activities with no high level of daily activities, such as jumping and jogging after 6 weeks postoperatively. 27 patients were available for follow-up for at least 2 years, while only one patient was lost to follow-up. At postoperative 2 years, the postoperative AOFAS score increased significantly, while the VAS score decreased statistically when compared with preoperative values. At final follow-up, 24 patients had complete alleviation of pain, whereas the remaining 3 patients complained of mild heel pain after walking for a long time. The MOXFQ score showed obvious relief of previous symptoms for all included cases.

**Conclusions:**

Central tendon-splitting approach and double row Achilles tendon suture provide excellent intraoperative visual field, larger tendon-bone contact area, and stronger pullout strength and, thus, facilitate early rehabilitation. It can be a safe and effective method for the treatment of insertional Achilles tendinopathy.

## 1. Introduction

Insertional Achilles tendinopathy is characterized by chronic refractory posterior heel pain associated with posterosuperior calcaneal exostosis or intratendinous ossification. The etiologies include retrocalcaneal exostosis, the diseased tendon, enthesopathy, and Haglund's deformity. Its histological changes include increase in the number of tenocytes, concentrations of glycosaminoglycans in the ground substance and collagen fragmentation. The nonsurgical therapies, such as physical therapy, stretching and strengthening of the gastrocnemius-soleus muscle complexes, nonsteroid anti-inflammatory drugs, and footwear modifications, are chosen as the initial treatment. Local injection of the steroid is not recommended because it might lead to increased risk of acute tendon tear [1–3]. The surgical intervention is indicated if the nonsurgical therapies is failed for more than six months. Surgical interventions are performed using either open or endoscope method. Endoscopic calcaneoplasty (ECP) is used as a minimally invasive method to debride the exostosis prominence [4]. However, sufficient debridement of retrocalcaneal exostosis may not be achieved, moreover, intratendinous calcification and the full-thickness intratendinous lesions cannot be deal with by ECP [5]. Recently, reports on endoscopic calcaneoplasty for insertional Achilles tendinopathy are few [6]. Open surgical approach can avoid the drawbacks of endoscope method. There are several open surgical approaches, such as medial J-shaped incision, [7] lateral incision, transverse incision [8], double incision and central Achilles tendon splitting incision. However, no study has concluded that one approach is superior to another in the management of insertional Achilles tendinopathy so far. In this preliminary study, we aim to evaluate the efficacy of the central tendon-splitting approach and double-row Achilles suture bridge technique for surgical management of insertional Achilles tendinopathy. Also, we will discuss some associated precautions.

## 2. Patients and Methods

### 2.1. General Data of Patients

From January 2014 to November 2016, 28 patients with hindfoot pain (28 feet), including 17 males and 11 females, with the mean age of 43 years (ranging from 26 to 58 years) were diagnosed as the insertional Achilles tendinopathy. The majority of patients were actively involved in sports, such as badminton, basketball, tennis and running. Some of the patient had high BMI level companied with high level of uric acid in blood. Before the operation they all received one or more non-surgical treatments such as calf stretching, nonsteroidal anti-inflammatory drugs, extracorporeal shock wave therapy or heel lifts. Steroid injections were not used because of the concern of Achilles tendon rupture. The inclusions are as follows: (1) symptom of hindfoot pain, (2) radiographic demonstration of calcifications around the insertion of the Achilles tendon or degeneration of the Achilles tendon, (3) no improvement after standard nonsurgical treatment for more than six months. Exclusion criteria were use of an alternative surgical technique for insertional Achilles tendinopathy or refusal to accept surgical treatment. Regarding physical examination, tenderness was elicited when pressing the Achilles tendon insertion zone, and aggravated when performing plantar flexion. A thickened distal Achilles tendon was also found. Moreover, the muscle strength of plantar flexion was decreased when compared with the contralateral leg. 22 cases could not perform the heel-rise test well due to the hindfoot pain.

### 2.2. Radiographic Assessment

**Figure 1:**
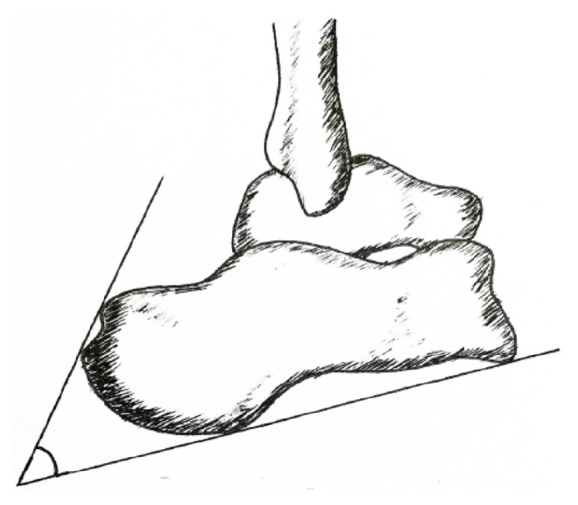
Diagram of Fowler-Phillip angle.

**Figure 2:**
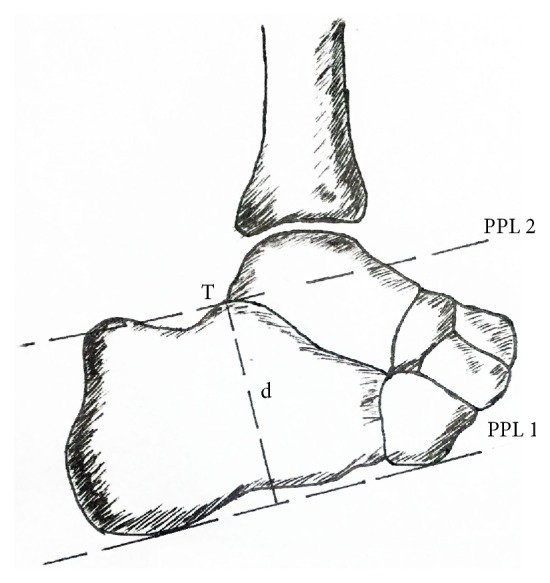
Diagram of parallel pitch line.

Preoperative lateral X-ray was taken to check the posterior superior calcaneus exostosis (Haglund deformity) and tendon calcification. The normal Fowler-Phillip angle (FPA) ranges from 44° to 69° [9, 10]. If FPA is more than 75°, it is suspected to have an enlargement of the posterior aspect of the calcaneus (Figure 1). The bursal projection touching or below the Parallel Pitch Line 2 (PPL2) is normal (Figure 2). Magnetic resonance imaging (MRI) was used to evaluate bursitis, edema, and thickening of the distal portion of the Achilles tendon.

### 2.3. Surgical Technique

Patients were placed in the prone position. A padded thigh tourniquet and sterile draping was applied. A longitudinal slight inward incision over the posterior heel using the central tendon-splitting approach was performed, which was slightly different from what Mc Garvey et al. [11] described.

The Achilles tendon was split through the central portion and detached slightly from its insertion on the calcaneus. About 60% to 70% of the tendon insertion area was detached. Once the split tendon flaps were lifted on the sides, the tendon bursae and posterior calcaneal protrusion were exposed. Then, any degenerative or calcified lesions were debrided. The retrocalcaneal bursa was resected thoroughly until healthy tissues could be seen. An oscillating saw was used to resect the prominence of the exostosis. Intraoperative fluoroscopy was used to confirm whether sufficient bone spur had been resected. The ankle was dorsiflexed to confirm whether there was any residual impingement between the tendon and the posterior calcaneal protrusion. Following this, a proper flat bony bed surface was prepared. Next, the tendon flaps were lifted to both sides, and two inner up row anchors (4.5 mm anchors, DuPuy Synthes, New Brunswick, NJ) were inserted. These two inner holes were made by the 3.2 mm drill approximately 1.5 cm proximal to distal insertion of the tendon footprint. Then, the inner anchors were inserted. Another two outer low anchors (4.5-mm anchors, DuPuy Synthes, New Brunswick, NJ) were inserted 0.5-0.8 cm distal to the distal aspect of tendon footprint lying parallel and inferior to the inner anchors. Then the tendon was reattached using a modified double-row Achilles suture bridge technique. The double-row and crisscross configuration were made to tighten and reattach the tendon to its insertion site.

**Figure 3:**
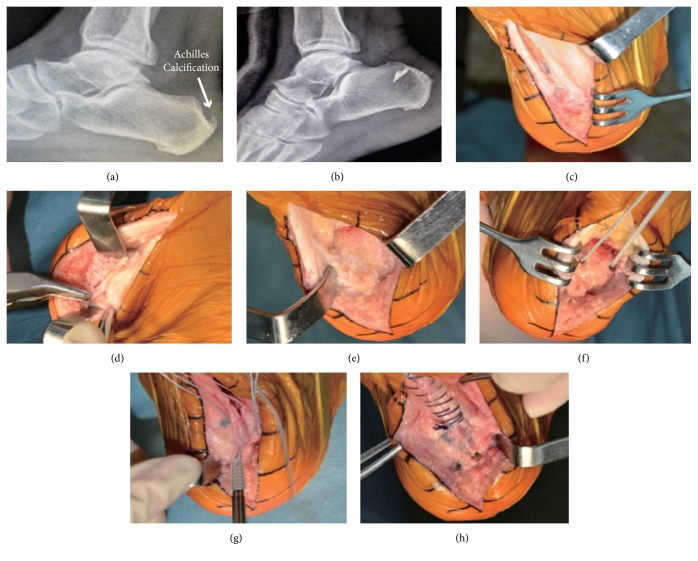
*Case 1, a 67-year-old male complained of right posterior heel pain for 1 year. He could not perform the heel-rise test preoperatively and also showed uric acid level of 462 mmol/L in blood*. (a) Preoperative radiograph showed calcification in the Achilles tendon insertion. (b) Postoperative radiograph demonstrated that calcaneal calcification had been removed. (c) Central tendon-splitting approach through longitudinal incision was used to partially elevate the tendon. (d) The tendinosis portion was thoroughly debrided, and the exostosis was removed by rongeur forceps and oscillating saw. (e) After the exostosis were completely resected, the posterior calcaneal wall was levelled off. (f) The inner anchors were inserted in the proximal calcaneal tuberosity. (g) The outer anchors were inserted in the distal calcaneal tuberosity and formed the double row suture bridge. (h) Continuous suture was used to repair the split tendon.

**Figure 4:**
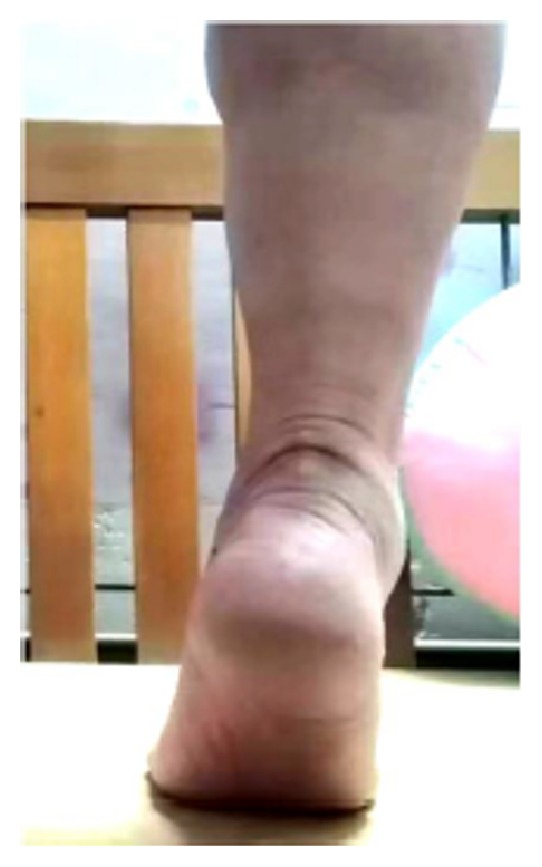
The patient of case 1 performed the heel-rise test at the final follow-up.

**Figure 5:**
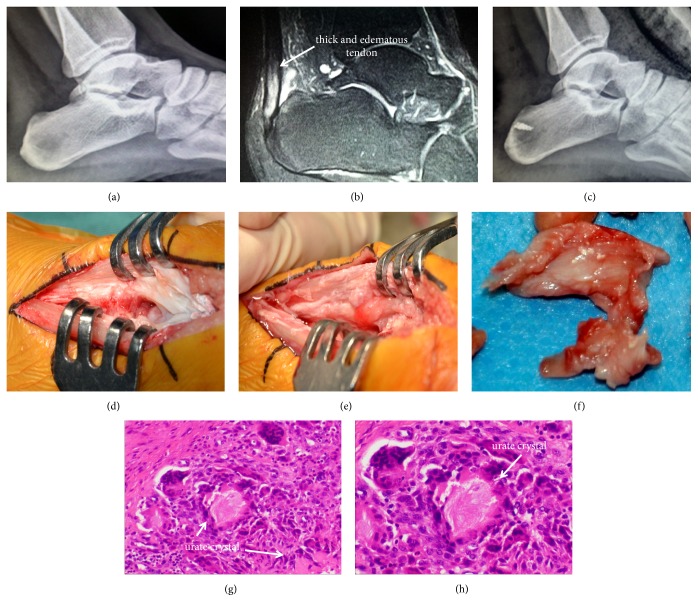
*Case 2, 24-year-old male complained of right posterior heel pain for 2 years with blood uric acid of 692 mmol/L*. (a) Preoperative radiograph showed calcification of calcaneus in the Achilles tendon insertion. (b) Preoperative MRI found edematous and thickened Achilles tendon, posterior calcanues bursitis and edema in the posterosuperior calcaneus. (c) Postoperative radiograph showed calcaneal calcification had been removed and anchor position was satisfying. (d) Central tendon-splitting approach was used to perform the surgery. (e) After splitting the Achilles tendon, the denaturation tendon could be visualized. (f) The removed denatured paratendon tissue. (g)-(h) The lesion tissue was stained by haematoxylin and eosin. Under the light microscope (100× and 200×), infiltrating neutrophils, lymphocytes were found, and the white arrow showed multinucleated giant cells in different morphologies and sizes surrounding the urate crystal.

**Figure 6:**
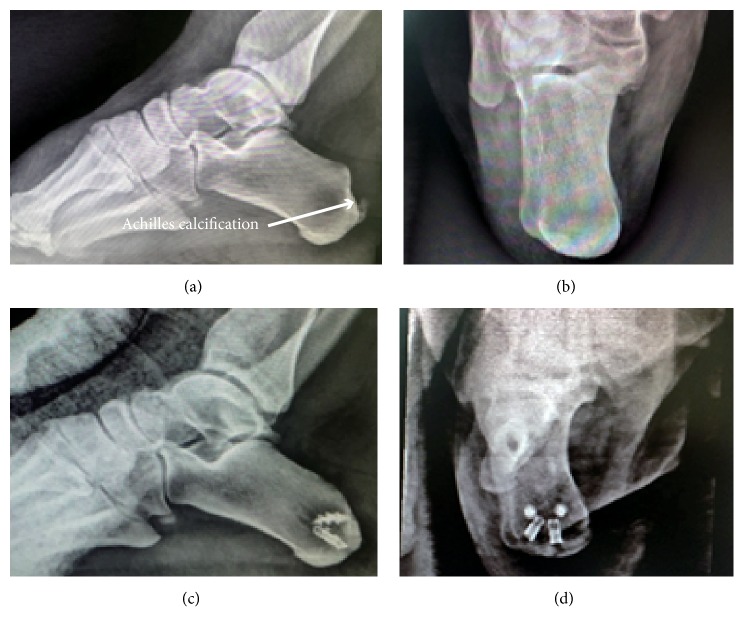
*Case 3, a 40-year-old woman showed right posterior heel pain for 3 years, and her nonsurgical treatment was ineffective*. (a) Preoperative radiograph revealed calcification near the posterior calcaneus. (b) Axis X-Ray plain film of calcaneus before operation. (c) Postoperative radiograph showed calcification in the Achilles tendon insertion had been removed, and the Achilles tendon was fixed by double row suture bridge. (d) Postoperative calcaneal axis X-Ray film showed the distribution of inner and outer anchors.

The flexor hallucis longus (FHL) transferring procedure could be considered if the patient aged more than 50 years or with severe degeneration of tendon. [6] However, we did not apply this procedure in our cases. Figures 3–6 list the typical cases of 3 patients.

Finally, the wound was thoroughly irrigated and sutured. The operated foot was immobilized with plaster in a plantar flexion position. The resected tissues were stained and analyzed under microscopy.

### 2.4. Postoperative Rehabilitation

A short leg plaster cast was applied for 4 weeks in slight plantar flexion position postoperatively. After removal of the plaster, the rehabilitation activities, such as gastroc-soleus stretching, partial weight-bearing with a walking-boot were initiated. Passive dorsiflexion, active resistive plantar flexion, plantar inversion and eversion were performed. Then a 1.5-2 cm felt heel lift Achilles tendon boot which make the plantar flexion about 20-30° was used to alleviate the pressure on the insertion site. Partial weight-bearing activity was progressively increased until achieving full weight-bearing at 6 weeks postoperatively. Step by step the felt heel lift was changed to a lower height. Finally it was totally removed and change to normal shoes. High level of daily activities, such as jumping, jogging, and deep crouching were suggested to gradually start at 12 weeks postoperatively. Strenuous sports were not recommended until 6 months postoperatively.

### 2.5. Parameter Assessments

The visual analog score (VAS) was used to measure pain intensity. Zero points indicated no pain, while ten points indicated extremely severe pain. The American Orthopaedic Foot and Ankle Society (AOFAS) Ankle-Hindfoot scale was used to evaluate the functional outcomes [12] (Table 1). It includes pain (40 points), function (50 points) and alignment (10 points). At final follow-up, patient-reported outcome measures (PROMs) in the form of Manchester-Oxford Foot Questionnaire (MOXFQ) [13–15] was collected to evaluate clinical outcome. MOXFQ scale contains 16 items, each item is scored from 0 to 4, with 4 representing the worst stage. The scores can be converted to a 0 -100 scale using the formula (summation/64*∗*100).

### 2.6. Statistical Analysis

Statistical analysis was carried out by using the SPSS software (IBM, version 21.0). VAS Score, AOFAS ankle-hindfoot scale before and at 2 years after the surgery were expressed as median and interquartile range. FPA were expressed as the mean ± standard deviation. The preoperative and postoperative VAS Score, AOFAS scale were evaluated by Wilcoxon signed rank test. The preoperative and postoperative FPA was evaluated by pair-t test. Statistical significance was set at *P* < 0.05.

## 3. Results

27 patients were followed up ranging from 24 to 32 months (average 27.3±2.1 months), while one patient lost follow up. Postoperative X-ray showed that the calcaneal spur was thoroughly debrided and the anchors were in satisfactory position. At final follow-up, significant improvements of VAS, AOFAS ankle-hindfoot scale and FPA were found when compared with preoperative values (Table 2). We considered the minimal important change (MIC) values for AOFAS and the VAS were 15 and 3, respectively. 24 patients had complete alleviation of pain, whereas the remaining 3 patients complained of mild heel pain after walking for a long time. MOXFQ score was 18.8±7.2 (9.4 to 35.9) at the final follow-up. No perioperative complications, such as wound infection, hypoesthesia around the incision site, scar irrigation or nerve injury were observed. Of the 27 patients, 26 cases could perform the heel-rise test at the final follow-up. Three patients had a loss of ankle dorsiflexion by approximately 10°, while one had about 15° loss.

## 4. Discussion

### 4.1. The Value of FPA and PPL

The FPA and PPL were used to quantify the bone deformity, but they do not have a definite relationship with reported symptoms. In this series, the average FPA was 58.9±4.9°, which was less than 75° and the bursal projection in most cases was below the PPL_2_. Due to the fact that insertional Achilles tendinopathy has both the bony and soft tissue abnormalities, X-ray measurement is not enough to evaluate the insertional Achilles tendinopathy. Lu et al. [16] also concludes that the FPA and PPL are just an ancillary for the decision to perform surgery, and surgical determination should be based on the clinical presentations of patients.

### 4.2. The Advantages of the Central Tendon-Splitting Approach

There are different approaches of managing the insertional Achilles tendinopathy surgically, including vertical J-shaped medial or lateral incision, double incision, transverse Cincinnati incision and central Achilles tendon splitting incision. However, no consensus has been reached regarding the best one. The medial or lateral incision approach is known to be less damaging to the Achilles tendon, but these approaches do not provide sufficient exposure. Maffulli et al. [17] reported outcomes using transverse Cincinnati approach, in which 2 out of 30 cases have superficial wound infections. The central Achilles tendon-splitting approach, which was first reported by Nunley et al. [18] in 2011, appears to be superior to others due to various advantages [7, 19]. This approach provides the best exposure to the Achilles tendon. All procedures are performed under direct visualization. Therefore, a rapid and thorough debridement for all lesions can be achieved, and postoperative resolution of the symptoms would be promising. Moreover, risks of injury to sural nerve, posterior tibial vessels and branches of peroneal artery are minimal. No neurovascular complication is found in our study. Lastly, the FHL transfer can be easily carried out if needed. To reduce the possible postoperative scar irrigation while wearing shoes and aesthetics of the incision, we make the skin incision slightly inward, which is slightly different from the previous report.

### 4.3. The Advantage of the Double Row Suture Bridge Tendon Suture

**Figure 7:**
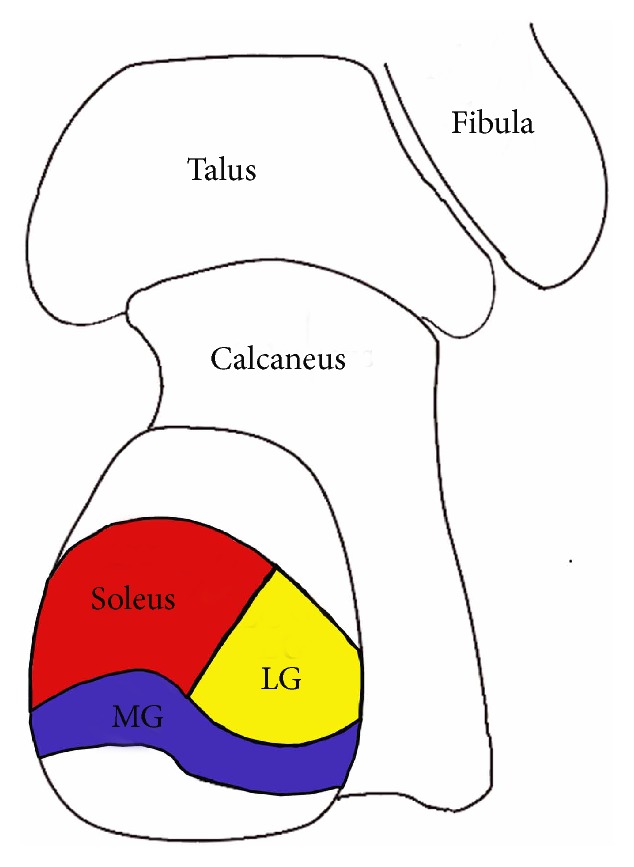
Illustration of the footprint of the medial head of the gastrocnemius (MG), the lateral head of the gastrocnemius (LG) and the soleus.

**Figure 8:**
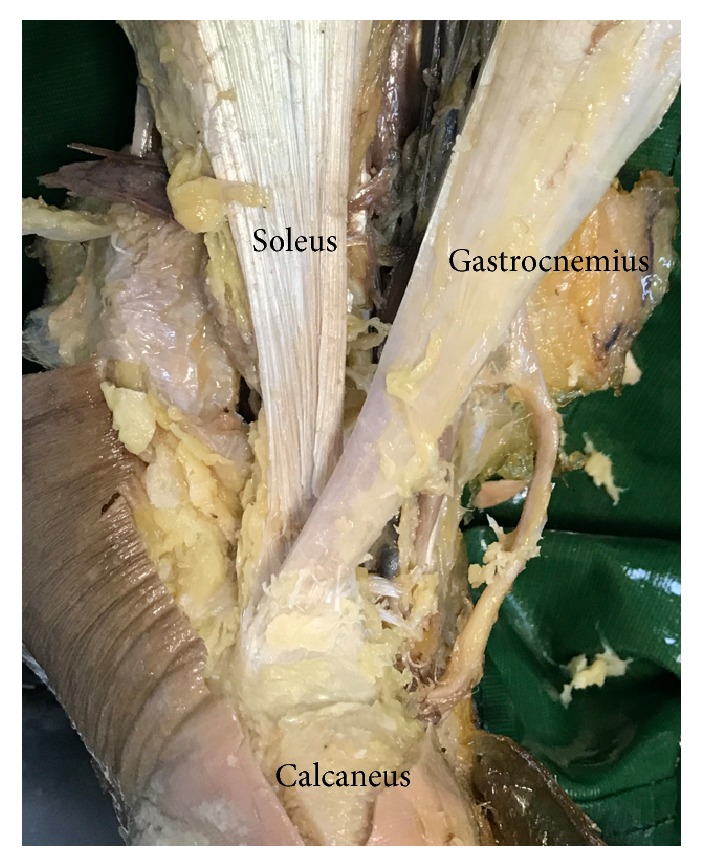
Cadaveric picture of anatomical footprint of the Achilles tendon.

It has been well understood that if less than 50% of the tendon has been detached, it can be repaired without suture anchors. However, if the detachment is more than 50%, reattaching the maximum possible tendon footprint is essential in the management of insertional Achilles tendinopathy [20]. Although different surgical methods have been used to augment the tendon, such as single row anchors and FHL transfer, we choose double-row suture technique to reattach the tendon. The double-row Achilles suture bridge technique has several advantages. Firstly, the double row suture bridge provides a larger area of contact between the Achilles tendon and bone, which can be beneficial to promote the healing. According to the report of Ballal MS et al. [21] based on the anatomical study of the Achilles tendon footprint, the insertion of the medial head of the gastrocnemius (MG), the lateral head of the gastrocnemius (LG) and the soleus are distributed in three parts on the calcaneus. The MG inserts into the inferior facet across the whole width of the calcaneal tuberosity. The mean width of the footprint is 28.3 mm (24 to 34 mm), and its mean length is 7.8 mm (6 to 10 mm). The footprint of LG is in lateral part of middle facet with mean width of 14.4 mm (12 to 19 mm) and mean height of 10 mm (7 to 12 mm). While the footprint of soleus is in medial part of facet with mean width of 18.2 mm (15 to 28 mm) and mean height of 15 mm (13 to 17 mm) (Figures 7 and 8). Thorough debridement of the Achilles tendon lead to detachment of the MG, LG and soleus muscle footprint in different levels. This is the anatomical proof that supports the use of double row suture, which can ensure better reattachment of the three different components of the Achilles tendon to the calcaneus than the single row suture. As we know, this advantage has not been reported in previous studies. Secondly, the double row suture can provide stronger pullout and fatigue strength leading to a lower rate of tendon rupture. The tendon quality of most Achilles tendinopathy patients is poor. Theoretically, for patients with extensive tendon debridement, the double-row Achilles suture bridge technique have the biomechanical advantage over the single-row anchor sutures [22]. Jeffrey E et al. [23] report a retrospective review of 98 patients (100 feet) who have undergone Achilles reattachment following insertional Achilles debridement. Four patients (4.0%), who show BMI that is greater than 28 kg/m^2^ and in whom 1 or 2 anchors have been used, require revision or repeat repair because of failure of the anchor or tendon avulsion. By contrast, those using four anchors do not have the anchor failure.

**Figure 9:**
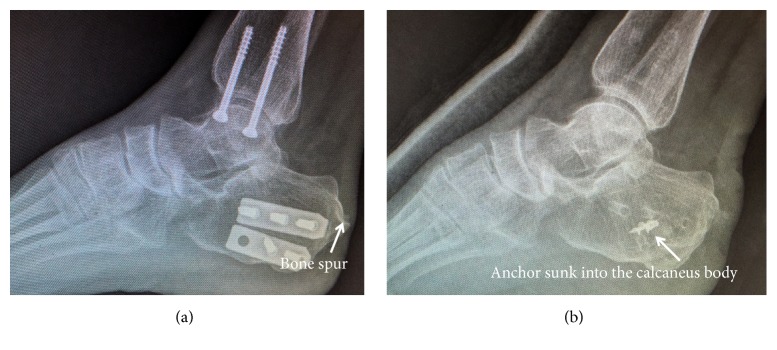
*Case 4, a 61-year-old woman showed right posterior heel pain for 1 year, and she also underwent calcaneal and medial malleolus medialis fracture 1 year before the symptom*. Due to osteoporosis and inappropriate force during operation, the anchor sank into the calcaneal body, causing the decreased pullout strength of anchor. (a) Preoperative radiograph showed the bone spur near the posterior calcaneus and osteoporosis sign. (b) Postoperative radiograph demonstrated the anchor had sunk into the calcaneus body.

Furthermore, some old patients may have poor bone density, which decrease the pullout strength of the anchor. An unsatisfied case of 60 year-old female (Figure 9) (not included in this case series) used one anchor to reattach the Achilles tendon. However, due to osteoporosis and the use of inappropriate force intraoperatively, the anchor sank into the calcaneal body, which may cause the decreased pullout strength of anchor. At last, more and more patients in modern day society required to return to their original jobs and life as soon as possible. The double row suture bridge can facilitate their postoperative recovery, especially for those with high BMI or active activity [24, 25]. Due to the nature of the double-row suture, applying a cast below the knee is sufficient postoperatively. The plaster immobilization time is shortened, benefiting the earlier initiation of functional rehabilitation. Therefore, ankle stiffness, gastrocnemius muscle atrophy, and the plantar flexor muscle strength deficit can be prevented. Another 40 year-old female patient, who shows BMI of 30.71 kg/cm^2^ (Figure 6), has the satisfactory clinical outcomes. Besides, the double row suture bridge technique reduces the prominent bulky suture knot and thus, there is less discomfort while wearing shoes.

### 4.4. Precautions during Surgery

The extent of the Achilles tendon stripping should not exceed 70% of the insertion area, and the complete detachment of the Achilles is seldom required. Nunley et al. [17] report up to 70% of the tendon insertion area can be safely detached to visualize the entire distal spur with no cases of tendon rupture. Secondly, insufficient debridement of Achilles tendon lesions may affect postoperative outcomes. Hardy A et al. [26] report a retrospective analysis of 46 patients who receive surgical treatment for insertional Achilles tendinopathy. Poor symptom relief is observed in all patients with less than 50% tendon involvement, while the cases undergoing detachment/reattachment have satisfactory functional outcomes. He speculates that this may be due to the incomplete intraoperative assessment of the tendon lesions or non-exhaustive debridement of the lesion. Thirdly, we find that Asian people, especially women, have smaller area of the calcaneus heel. Hence, anchor screws with smaller diameter should be selected for these patients, and it requires to be positioned moderately depending on the detachment area of tendon footprint. Precautions must be taken while drilling the holes for the anchors on the calcaneus. The drill should be angled towards the midline and leave a distance from the lateral and medial walls to prevent the disruption of the cortical wall.

### 4.5. Achilles Tendinopathy with Hyperuricemia

Among the total 28 cases in our study, there are 10 patients accompanied by hyperuricemia. Uric acid crystals deposition in the tendon can cause low-grade persistent inflammation and accelerate the degeneration of the tendon [27]. Sarah Stewart et al. [28] report that formation of gouty tophi is associated with force deficits and might contribute to reduced muscular activity and disuse muscle atrophy. During the acute gout attack, these patients tend to have more pain in the hindfoot. In case 2 (Figures 5(g) and 5(h)) different sizes of multinucleated giant cells infiltrating surrounding the urate crystals in the excised tissue can be seen under the microscope. This phenomenona is in accord with the increasing incidence of hyperuricemia among the younger population in China [29]. Because the gouty crystal salt is extensively deposited in Achilles tendon, extensive detachment of the Achilles tendon should be performed in these patients to achieve complete lesion eradication. Central tendon-splitting approach and double row suturing method is suitable for these specific patients to achieve firm fixation and early rehabilitation. Moreover, it is also essential to reduce the level of uric acid postoperatively to ensure better clinical outcomes for patients with hyperuricemia.

### 4.6. The Limitations of This Study

There were some drawbacks in this study. Firstly, this is merely a retrospective study with limited included cases. Secondly, only one group was established in this study with no comparison groups receiving nonsurgical treatment or other surgical methodologies. Thirdly, MOXFQ score at final follow-up was obtained and there was lack of its preoperative assessment. Therefore, a more comprehensive prospective study with larger sample size and more assessments should be initiated in the future.

## 5. Conclusion

Considering the increased proportion of poor Achilles tendon texture in the insertional Achilles tendinopathy patients and the need of rapid functional recovery postoperation, the central tendon-splitting approach and double row suture bridge tendon suture is convenient for thorough debridement and firm fixation of Achilles tendon with low complication incidence. Therefore, it is an effective surgical option for the insertional Achilles tendinopathy.

## Figures and Tables

**Table 1:** The American Orthopaedic Foot and Ankle Society (AOFAS) Ankle-Hindfoot scale.

AOFAS Ankle Hindfoot Scale	score
*pain*	
none	40
Mild, occasional	30
Moderate, common	10
Serious, continuous	0
*Functional and autonomous activities and support*	
Unlimited, no need support	10
Daily activities are not limited, recreational activities are limited, handrails are required	7
Daily and recreational activities are limited, requiring handrails	4
Daily and recreational activities are severely restricted and need support cars, aides, wheelchairs, stents	0
*Walking distance (number of blocks)*	
>6	5
4-6	4
1-3	2
<1	0
*Ground walk *	
No difficulty on any ground	5
Difficulties in walking uneven floors, stairs, slopes, and ladders	3
Difficult to walk unevenly on the ground, stairs, slopes, and ladders	0
*Abnormal gait*	
None, slight	8
obvious	4
Significant	0
*Activities (flexion and extension)*	
Normal or mildly restricted (>30°)	8
Moderately restricted (15°-29°)	4
Severely restricted (<15°)	0
*Hind foot activity (inversion and eversion)*	
Normal or mildly restricted (75%-100% normal)	6
Moderately restricted (25% -74% normal)	3
Severely limited (<25%)	0
*Ankle-hind foot stability (front, back, varus - valgus)*	
stable	8
Obvious instability	0
*Foot alignment*	
Excellent: foot, ankle-hind foot alignment normal	10
Good::foot, ankle-hind foot alignment obviously angulation, asymptomatic	5
Bad: Severely disordered, symptomatic	0

Excellent: 90-100; good:75-89; acceptable:50-74; bad: below 50.

**Table 2:** Improvements of clinical outcomes after surgery (n=27).

	Preoperative Mean Score	Follow-up Mean Score	*P* value
VAS score	(6,7)	(0,1)	<0.001
AOFAS ankle-hindfoot scale	(48,61)	(92,98)	<0.001
FPA	58.9±4.9	50.1±4.4	<0.001

## Data Availability

The data used to support the findings of this study are available from the corresponding author upon request.

## Abbreviation

AOFAS: American Orthopaedic Foot and Ankle Society
VAS: Visual analogue score
PPL: Parallel pitch line
FHL: Flexor hallucis longus
FPA: Fowler-Phillip angle
ECP: Endoscopic calcaneoplasty
MOXFQ: Manchester-Oxford Foot Questionnaire
MRI: Magnetic resonance imaging
MG: The medial head of the gastrocnemius
LG: Lateral head of the gastrocnemius.
